# Future scientific publishing: what should be inherited and changed?

**DOI:** 10.1093/nsr/nwz224

**Published:** 2020-02-24

**Authors:** Zhonghe Zhou

**Affiliations:** 1 Institute of Vertebrate Paleontology and Paleoanthropology, CAS, China; 2 Associate Editor-in-Chief of NSR

The Chinese government has recently announced new polices to improve its publishing ecology and increase the support for developing world-class scientific journals, including those in Chinese, in order to achieve a higher profile in the world academic community. This is an ambitious and yet challenging mission. However, it also seems a due move in view of the current debate and uncertainty, as the world's scientific publishing is moving towards a digital and open-access age. Scientific publishing has changed dramatically over the past 350 years and is now facing unprecedented challenges: what should be inherited and changed?

Over the past centuries, the four basic principles or roles of scientific publishing, i.e. scientific priority (registration), peer review (certification), archiving (preservation) and dissemination, have become the foundations for nearly all scientific journals today. Scientific priority continues to be a core value in scientific research and publishing helps to establish the priority of the discovery of research. In an age of growing expansion of research output and fast dissemination of information, peer review proves to be a valuable concept. However, with the outburst of new disciplines and enormous data for supplementing the main text, it also poses a challenge for journal editors to obtain sufficient reviews from qualified referees in due time. It is also notable that archiving and dissemination have become more and more speedy and convenient, thanks to the development of digital technology.

English is currently the global language of scientific publishing and continues to spread and expand its impact in more countries and on social and human sciences. However, the dominance of English in scientific communications also brings up the worry of a loss of diversity in scientific-publishing ecology, as many institutions and universities are under the pressure of ‘internationalization’ often signified by publishing in English. Scientific publications in non-English languages should be protected in order to encourage research and publications on issues focusing on regional or cultural specificities. It is often ignored that scientific publications in local languages can play an important role in scientific communications, policymaking and science education. Thus, the existence of many unique-niche journals is critical not only for the development of local research cultures and societies, but also for the global scientific community as well.

The impact of the digital age on our life is greater than at any time in human history. The transformation of current publishing modes is ongoing and under frequent discussion. The advantage of digital publications is obvious, but is it necessary to exclude traditional print publishing? The answer is at least not self-evident today. The strict peer-review system has sometimes been blamed for impeding publications of contentious or truly revolutionary ideas, and authors tend to compromise their opinions to secure publications in high-profile journals. Thus, publications of more debates or critiques are necessary to offset the effect of pressure from peer review. It is also a good idea to publish controversial yet potentially important papers with referees’ comments together. It seems that more publishing modes will be tried in the near future.

Throughout the history of scientific publishing, the scientific community has been collaborating with the publishers. It is almost sure that the academic publishing industry will continue to exist despite the current complains from the scientific community. However, the situation of a few mega publishers controlling the scientific-publishing market has indeed incurred criticisms. Although the concept of an impact factor was itself neither created by the publisher nor designed to measure the research quality of a researcher or a research institution, it is an undeniable fact that the mega publishers and high-profile journals are less concerned about the negative effect of the impact factor or indices on how scientific research is fairly assessed. Thus, the scientific community should take the responsibility to make a change.

The recently growing appeal for open-access publishing for more effective and productive dissemination of scientific ideas represents another major debate between the scientific community and publishers. Science is an inseparable part of society and the impact of scientific progress on social and economic development is becoming more and more significant. Mega publishers must realize that, as their success has been based on scientific activities, they are obliged to adapt to the future development of scientific publishing. On the other hand, in an age of global economy controlled by the market, the scientific community cannot claim a victory alone without cooperating with publishers. Hopefully, such a debate that is itself due to technological progress will be solved by science administration to push scientific publishing forward in the direction that benefits human societies.

